# Remote Patient Monitoring Across European Health Systems: Policy Insights from a Cross-Country Stakeholder Study

**DOI:** 10.3390/healthcare14193147

**Published:** 2026-09-22

**Authors:** Evangelia Salmatani, George Dimitriou, George E. Dafoulas, Dimitrios G. Katehakis, Apostolia Karampatea, Maarten Gijssel, Dorota Waclawczyk, Diana Vertelkienė, Vasiliki Zilidou, Styliani Olympia Tsormpatzoudi, Maria Mavridi, Isabel María Díaz Lozano, Maria Cuartero, Gastón A. Crespo, Kęstutis Morkūnas, Angelina Kouroubali

**Affiliations:** 1 Hellenic Digital Health Cluster, 105 59 Athens, Greece; dimitriou@hdhc.gr (G.D.); gdafoulas@med.uth.gr (G.E.D.); katehaki@hdhc.gr (D.G.K.); kouroub@ics.forth.gr (A.K.); 2 Institute of Computer Science, Foundation for Research and Technology Hellas, 700 13 Heraklion, Greece; 3 Gnomon Informatics SA, 570 01 Thessaloniki, Greece; a.karabatea@gnomon.com.gr; 4 IDRO B.V., Bd de Waterloo 65, 1000 Bruxelles, Belgium; maarten@idro.world (M.G.); dorota@idro.world (D.W.); 5 i-Vita Health Technology Cluster, LT-47175 Kaunas, Lithuania; diana@defuturo.lt (D.V.); kestutis@defuturo.lt (K.M.); 6 Lab of Medical Physics & Digital Innovation, Aristotle University of Thessaloniki, 541 24 Thessaloniki, Greece; vickyzilidou@gmail.com (V.Z.); stellatsormpatzoudi@gmail.com (S.O.T.); mariaphys2000@gmail.com (M.M.); 7 UCAM-SENS, UCAM HiTech, Universidad Católica San Antonio de Murcia, Avda. Andrés Hernández Ros, 1, 30107 Murcia, Spain; imdiaz@ucam.edu (I.M.D.L.); mariacuartero@ucam.edu (M.C.); gacp@kth.se (G.A.C.)

**Keywords:** remote monitoring, telemedicine, health policy, healthcare innovation, wearable electronic devices, vital signs tracking, chronic disease management, mobile health, remote consultation, self-care

## Abstract

**Highlights:**

**What are the main findings?**

- Within the country contexts examined, stakeholders more frequently identified systemic conditions, including governance, reimbursement, interoperability, and workforce readiness, than technology availability alone as barriers to RPM implementation and scale-up.
- The country contexts examined showed variation in the reported integration of RPM into care pathways: some stakeholders described comparatively more established implementation, while others reported activity concentrated in pilot initiatives, with limited scale-up and fragmented adoption.

**What are the implications of the main findings?**

- The findings suggest that scaling RPM would benefit from coordinated policy action, including more sustainable reimbursement models, clearer regulatory pathways, and stronger interoperability with electronic health records (EHRs).
- The implementation and potential impact of European initiatives such as the European Health Data Space (EHDS) are likely to be influenced by national implementation capacity and the readiness of the participating health ecosystems.

**Abstract:**

**Background/Objectives:** Remote patient monitoring (RPM) is recognized as a key component of health system transformation in Europe, in the context of vulnerable populations. However, its large-scale adoption remains uneven across countries. The research explored how technical infrastructure, governance and regulation, interoperability, and ecosystem readiness influence RPM adoption and scale-up in seven European country contexts. **Methods:** An exploratory qualitative cross-country study combined targeted desk research with semi-structured interviews conducted between February and March 2025. The interviews were recorded with consent, manually transcribed, anonymized, and analyzed using a manual hybrid deductive–inductive thematic approach. The desk research provided contextual information and remained distinct from the interview-derived findings. **Results:** The analysis was organized around four higher-order themes including digital infrastructure and RPM technologies; governance and regulatory frameworks; interoperability and health-data integration; implementation barriers and ecosystem readiness. RPM technologies were present in all examined contexts, but their integration into routine care remained uneven. Recurrent barriers included fragmented reimbursement pathways, uneven operationalization of EU rules, limited interoperability, weak integration with Electronic Health Records and clinical workflows, digital-literacy gaps, and insufficient mechanisms for progressing from pilots to sustained implementation. **Conclusions:** Stakeholders more frequently identified system-level implementation conditions, including governance, reimbursement, interoperability, and workforce readiness, than technology availability alone as barriers to RPM scale-up. The European Health Data Space may provide an enabling framework, but its impact will depend on national and regional implementation capacity.

## 1. Introduction

European health systems are under growing pressure to respond to population ageing, multimorbidity, and chronic disease while also coping with workforce shortages and persistent inequalities in access to care. Recent European evidence shows that health system resilience increasingly depends on the ability to support healthy longevity, improve continuity of care, and make better use of digital tools and data infrastructures [1]. In this context, remote care has moved from being a peripheral innovation to a strategic component of health-system transformation.

Among remote care modalities, remote patient monitoring (RPM) has attracted particular attention because it enables the continuous or periodic collection and transmission of physiological and patient-reported data from outside conventional clinical settings [2]. Remote/client patient monitoring (RPM), also known as telemonitoring, “enables health workers to monitor an individual’s condition remotely, using technologies such as connected medical devices and sensors, often used for chronic conditions and accompanied by communication channels for coordination of care or alerts to health workers based on clinical parameters/ranges” [3].

RPM is especially relevant for long-term conditions, post-discharge follow-up, frailty, rehabilitation and home-based care pathways, where early detection of deterioration and timely clinical response may reduce avoidable healthcare utilization and improve care coordination [4,5,6]. Recent evidence suggests that RPM can improve safety and adherence outcomes and may contribute to lower readmissions, fewer outpatient visits, and reduced non-hospital costs in selected settings, although results remain heterogeneous relevant to conditions and implementation models [7,8,9]. RPM is a promising model whose value depends on clinical context, workflow design, and system readiness.

The rapid expansion of remote monitoring during and after the COVID-19 period accelerated this shift from experimentation to implementation [10]. At the same time, recent qualitative and review literature shows that the uptake of telemedicine and remote monitoring continues to be shaped by patient digital literacy, professional acceptance, usability, trust, and the extent to which technologies are embedded in real care pathways [7,11]. For chronic care in particular, telemedicine is most successful when it improves information quality, supports multidisciplinary coordination, and addresses the practical realities of everyday disease management rather than functioning as a stand-alone technical add-on [11].

At European level, the policy environment has also changed substantially. The European Commission’s recent analysis of wearable medical devices and digital health innovation highlights the growing role of connected technologies in enabling remote monitoring, hybrid care, and more patient-centred delivery models [12]. However, the same body of work also points to persistent barriers related to market access, fragmentation, certification, and deployment. Reimbursement remains one of the clearest examples of this unevenness. A recent cross-country policy mapping found that, although some form of digital health reimbursement now exists in several European countries, reimbursement pathways, pricing logics, and the classification of digital health solutions differ significantly [13]. These differences directly affect the scale-up potential of RPM and help explain why adoption remains uneven even where technological capability already exists.

Interoperability is another decisive issue. Recent evidence indicates that telemonitoring systems still rely on diverse and only partially harmonized technical standards. Although standards such as HL7 FHIR, ISO/IEEE 11073, and IHE profiles are increasingly visible in the literature, a universally adopted exchange model has not yet emerged, relevant to telemonitoring, to support interoperability across devices, providers, and cross-border contexts [14,15]. This has major implications for remote care deployment in Europe, where scaling RPM depends not only on the performance of devices or platforms, but also on their capacity to interact with Electronic Health Record (EHR), regional information systems, and future cross-border health-data infrastructures [16].

These developments underscore the importance of ongoing European regulatory efforts. The European Health Data Space (EHDS), adopted in 2025, establishes a common framework for access to, exchange of, and control over electronic health data in EU countries [17]. In parallel, EHDS has been shown to have the potential to strengthen research and innovation. However, this is not going to be possible unless trust issues, implementation capacity, and national readiness gaps are properly addressed [18]. The Artificial Intelligence (AI) Act is adding to the governance of high-risk digital health applications, including AI-enabled tools that will become increasingly relevant to RPM workflows [19]. Although the EHDS supports a transition from fragmented digital health initiatives toward a more structured European data and regulatory architecture, it does not by itself resolve persistent differences in national implementation capacity, interoperability, and digital maturity [20,21,22].

While the literature on telemedicine and RPM is expanding rapidly, much of it remains focused on specific diseases, technologies, or single-country experiences. There is still a limited understanding of how RPM ecosystems evolve across various contexts, and especially how governance, reimbursement, interoperability, and data readiness interact to support or constrain implementation. This gap is particularly important for countries and regions where digital health capacity is emerging but not yet consolidated.

To address this gap, this exploratory cross-country study examines the system-level conditions shaping RPM adoption and scale-up across seven European country contexts, through a multinational stakeholder study conducted within the IRHIS project (https://www.i-vita.lt/en/project/irhis/, accessed on 22 June 2026).

It addresses three research questions: (1) What are the principal characteristics of the RPM ecosystems examined? (2) How do governance, regulation, reimbursement, interoperability, health-data integration, workforce readiness, and ecosystem coordination enable or constrain implementation? (3) What policy and implementation priorities could support the transition from localized pilots to sustainable routine care? The study’s contribution lies in providing a cross-country, system-level perspective based on stakeholder accounts and complementary desk research. Unlike research focused on individual technologies, clinical applications, or single-country experiences, it examines how interconnected health-system conditions influence the capacity to implement RPM at scale.

## 2. Materials and Methods

IRHIS aims to accelerate the scale-up of remote health innovations and innovators offering AI-powered, cutting-edge sensor solutions, wearable devices, and remote monitoring solutions across three critical EU remote care areas: musculoskeletal rehabilitation, neurological disorder treatment, and critical-care monitoring, including detection of patient deterioration in intensive-care settings. Within the project, the partners conducted an exploratory qualitative multi-country study that combined semi-structured expert interviews with complementary targeted desk research to explore conditions affecting RPM implementation in the participating country contexts.

### 2.1. Study Design

The study was designed as an exploratory qualitative cross-country assessment of RPM implementation in selected European country contexts. It investigated stakeholder perspectives on the adoption and implementation of RPM services through semi-structured interviews, complemented by targeted desk research providing contextual information on relevant technological, regulatory, organizational, and health-system conditions. Qualitative inquiry is particularly appropriate for obtaining in-depth information from individuals with direct knowledge of complex and context-dependent phenomena, including emerging digital health ecosystems in which technological, regulatory, and organizational factors interact [21,22].

The study was conducted as part of the IRHIS project and focused on capturing insights from stakeholders actively involved in digital health innovation, healthcare delivery, and health policy. A semi-structured interview guide was developed to enable in-depth exploration of participants’ experiences and perceptions while maintaining sufficient flexibility to address country-specific developments and implementation challenges.

### 2.2. Participant Selection and Recruitment

The study was exploratory and policy-oriented. Participants were deliberately selected because of their relevant expertise and professional involvement in remote patient monitoring, digital health policy, healthcare delivery, regulation, research, or health innovation. This selection logic is consistent with purposive expert sampling, which seeks information-rich participants with direct knowledge of the phenomenon under investigation [23,24]. The stakeholder groups represented included healthcare providers, industry organizations, research institutions, governmental bodies, and European-level organizations.

Participant identification and recruitment were facilitated through the existing professional networks of the IRHIS consortium and its national partner organizations. The approach combined purposive expert selection with network-mediated and pragmatic recruitment. Participation was also influenced by stakeholder availability. No predetermined participant quota was established for each country, and the number of participants varied according to stakeholder availability and the structure of the respective national ecosystem. In some countries, one highly experienced stakeholder was interviewed because that individual held a leading role or had broad knowledge of national RPM developments.

The sampling approach aimed to obtain informed expert perspectives capable of describing important characteristics, opportunities, and challenges affecting RPM implementation in the participating country contexts. The resulting findings should therefore be interpreted as exploratory stakeholder perspectives rather than representative national assessments.

In total, twenty-two (22) stakeholders participated in the study. Participants represented a diverse set of professional backgrounds including chief executive officers, healthcare administrators, digital health advisors, and academic researchers. As Table 1 presents, the sample included stakeholders operating in multiple European countries, including Belgium, Bulgaria, Greece, Lithuania, Portugal, Spain, and the Netherlands, as well as representatives from European-level organizations.

### 2.3. Data Collection

Data collection was conducted between February and March 2025 through semi-structured interviews. Interviews were carried out virtually using secure online communication platforms, allowing participation from stakeholders located in different European countries. The interview guide was informed by the study objectives, the expertise of the research team, and selected sources concerning telemedicine implementation [3], digital health innovation [12], reimbursement [13], interoperability [14,15,16], and European regulation [17,19,25]. This contextual review was designed to highlight recurring implementation issues concerning technological infrastructure, governance and regulation, interoperability and data exchange, organizational integration, and the transition from pilot initiatives to sustainable implementation. These issues informed five domains in the interview guide: (i) the current state of RPM in the national context; (ii) the technical infrastructure and digital health platforms supporting remote monitoring; (iii) governance and regulatory frameworks affecting implementation; (iv) interoperability challenges and data-exchange practices; and (v) barriers and opportunities affecting the scale-up of remote monitoring services.

To enhance content validity and clarity, as well as to estimate the required interview duration, the guide was tested among project partners with relevant expertise. Feedback from this phase informed refinements to question wording, sequencing, and the use of prompts, improving both coherence and comprehensibility. In addition, the guide incorporated structured probing techniques to enable in-depth exploration of analytical themes, while ensuring consistency and comparability across interviews.

Interviews were conducted by a multidisciplinary team with expertise spanning remote patient monitoring, clinical practice, health informatics, data analysis, and digital health policy. To ensure methodological consistency and maximize the quality of insights gathered, all interviewers underwent a structured onboarding process that standardized interviewing techniques, established familiarity with the interview guide, and aligned expectations around adaptability to the natural flow of discussion, a key characteristic of semi-structured interviews.

Each interview lasted approximately 45–60 min. All participants were fully informed about the objectives of the study and provided informed consent prior to participation. With participants’ consent, the interviews were recorded and subsequently transcribed manually by members of the project team. No automated transcription software was used. The transcripts were anonymized before analysis by removing personally identifiable information.

The complete semi-structured interview guide, including the principal questions and prompts, is provided as Supplementary File S1.

### 2.4. Complementary Desk Research

Targeted desk research was conducted focusing on RPM implementation literature as well as digital health implementation in general in the EU domain and for each participating country. This work was undertaken, prior to stakeholder interviews, to provide contextual information and support the interpretation of stakeholder perspectives. Selected publicly available sources were reviewed, including national digital health strategies, legislation, policy documents, institutional and European reports, scientific publications, and relevant grey literature addressing RPM, telemedicine, reimbursement, interoperability, and digital health governance. The documentary information was considered alongside stakeholder accounts while remaining distinguishable from the interview-derived findings.

### 2.5. Data Analysis

Interview transcripts were analyzed manually using a hybrid deductive–inductive thematic approach [25]. Two researchers performed the initial review and coding of all interview transcripts. The coding was guided primarily by the domains covered in the study objectives and interview guide, while recurring observations within these domains were also recorded. A third researcher reviewed the preliminary codes and their organization into broader thematic categories. The resulting interpretations and thematic organization were discussed with the wider research team and finalized through discussion. Participants were informed that findings would be reported in aggregated form and that verbatim quotations would not be published. This was particularly important because some participants occupied senior or public-facing roles and because the small country-level samples could permit deductive identification.

The analysis was organized around four higher-order themes reflecting the study objectives and interview-guide domains, while incorporating recurring observations identified during coding. These themes formed the basis for the presentation and interpretation of the study findings. The analytical importance of a recurring observation was assessed in relation to the study objectives and its relevance across the stakeholder accounts, rather than solely according to its frequency of occurrence.

Additionally, the country-specific desk research was used to contextualize the stakeholder perspectives and the four analytical themes from the interviews. Given the exploratory design and uneven country-level sample, formal data saturation was not assessed or claimed.

During revision, the reporting of the qualitative component was reviewed against the Consolidated Criteria for Reporting Qualitative Research (COREQ) [26]. The completed checklist is provided as Supplementary File S2.

### 2.6. Ethical Considerations

The study adhered to established ethical standards for qualitative research. Participation in the interviews was voluntary. Informed consent was obtained from all participants prior to data collection. To ensure confidentiality and compliance with data protection regulations, all interview data were anonymized, and personally identifiable information was removed from transcripts.

The data collected for this study were used solely for research purposes and are reported only in aggregated form. Data management and storage procedures followed the data protection policies of the IRHIS project and complied with relevant European data protection regulations.

## 3. Results

An overview of the main emergent themes derived from the qualitative analysis and their corresponding sub-topics is presented in Table 2, which synthesizes the thematic findings. These are: (1) Digital infrastructure and RPM technologies; (2) Governance and regulatory frameworks shaping digital health adoption; (3) Interoperability and health data integration; (4) Implementation barriers and ecosystem readiness. The analysis of country-specific desk research and semi-structured stakeholder interviews across the seven countries’ contexts showed that RPM development in Europe is uneven and strongly conditioned by differences in digital maturity, regulatory clarity, interoperability capacity, and ecosystem support.

Stakeholders described variation in RPM integration and scale-up across the examined contexts, ranging from comparatively established implementation arrangements to activity concentrated in localized pilot initiatives. Across all settings, RPM was consistently viewed as strategically relevant for chronic disease management, ageing, rehabilitation, and continuity of care, but its uptake depended less on the existence of technology alone and more on the surrounding policy, financing, and organizational environment.

### 3.1. Digital Infrastructure and RPM Technologies

RPM technologies were already present in multiple forms at the stakeholders’ ecosystems, including wearable devices, app-based monitoring tools, telemedicine platforms, remote diagnostics, and AI-supported decision-support systems. RPM is addressed as an ecosystem combining wearable and implantable devices, cloud-based platforms, and teleconsultation tools that support continuous and patient-centred care beyond traditional clinical settings. It also notes that the technologies most commonly associated with RPM include patches, smartwatches, smart textiles, pulse oximeters, heart rate monitors, cardiac implantable devices, and continuous glucose monitoring systems, as indicated in Table 3.

However, the maturity of digital infrastructure varied considerably. Based on the stakeholders’ views, Belgium and the Netherlands were described as the most advanced environments. Belgium was described as having a rapidly evolving telemonitoring ecosystem supported by strong academic research, a large digital health startup base, living labs, accelerators, and the Belgian eHealth platform. The ecosystem included various companies and was characterized by app-based RPM, AI-powered clinical decision support, and a strong emphasis on integration with EHR. The Dutch stakeholder described a highly developed telemonitoring landscape, supported by major academic medical centers, strong public-private collaboration, and a mature startup ecosystem. This view was consistent with the findings of the accompanying desk research. Together, these sources suggest that RPM is embedded within broader digital health innovation ecosystems in both countries, rather than being confined to isolated pilot projects.

In the less developed settings, the technological picture was more mixed. Bulgaria had foundational digital infrastructure through the National Health Information System (NHIS), which supports e-prescriptions, EHR, and health data exchange, but its connection to advanced telemonitoring remained limited. The Bulgarian RPM landscape was described as still being shaped mainly by pilot projects led by private providers, startups, research groups, or local collaborations with hospitals and municipalities. Greece showed a similarly hybrid picture. Despite the existence of mature and technically sophisticated telemedicine platforms, including modular and device-agnostic systems integrating multiple diagnostic devices, EHR, video consultations, and AI-supported diagnostics, these were mostly deployed in localized pilots rather than on a national scale. Lithuania was described as actively developing an ecosystem centred on connected devices for chronic disease management, post-discharge monitoring, telemedicine platforms, and AI-assisted diagnostics, but with persistent infrastructure gaps. Spain and Portugal appeared comparatively more advanced among the less developed settings, with Spain benefiting from strong research and innovation environments in urban hubs and Portugal showing growing institutionalization of telemonitoring, including a documented portfolio of telemonitoring initiatives and visible implementations in rehabilitation and patient-centred telemonitoring.

A common finding across countries was that technical feasibility alone did not guarantee scale. In Greece, for example, interview findings suggested that platforms could already combine real-time monitoring, historical patient data, and video consultations, and could operate across multiple clinical domains, yet broader deployment was constrained by lack of a concrete reimbursement scheme and health-system integration. In Bulgaria, the main issue was not the absence of technological experimentation but the inability to move from fragmented pilots to formalized services. In Lithuania, promising startups and spin-offs existed, but their pathway into hospital workflows and wider health system use remained difficult. Spain and Portugal also demonstrated functioning digital solutions and visible innovation activity, but both countries still faced uneven implementation across territories and institutions.

### 3.2. Governance and Regulatory Frameworks

Governance and regulation emerged as one of the strongest differentiators between more mature and less mature RPM ecosystems. At the EU level, a broad regulatory environment relevant to RPM is identified, including the Medical Device Regulation (MDR) [27], In vitro Diagnostic Regulation (IVDR) [28], General Data Protection Regulation (GDPR) [29], the Data Governance Act [30], the Data Act [31], the AI Act—the world’s first comprehensive legal framework on AI, whose provisions are being applied progressively, with major requirements applicable from August 2026 [19], the EHDS [17], and the Cybersecurity Act [32]. At the same time, evidence from the analysis shows that these common European frameworks do not translate into uniform national implementation conditions. Instead, national and regional policy environments and regulatory frameworks differed markedly in how remote care was framed, enabled, or constrained. Belgium was characterized by regulatory complexity, fragmentation, and limited harmonization across national and regional levels. Although regional RIS3 strategies in Brussels, Flanders, and Wallonia strongly prioritize digital health, personalized medicine, and e-health, no clearly defined national legal framework specifically framing remote care was identified. Stakeholders considered Belgium both relevant and difficult as a test case because innovation was actively encouraged, yet reimbursement and regulation remained difficult to navigate. The national reimbursement system was repeatedly identified as a bottleneck, with only a limited number of digital tools successfully entering it. The Belgian case also showed incremental progress, including permitted teleconsultations under defined conditions and reimbursement schemes for selected mHealth applications.

The Netherlands differed from Belgium by being described as having a comparatively well-defined regulatory framework for digital health and telemonitoring. The Dutch environment was associated with clearer telemedicine guidance, reimbursement policies for digital health tools, and a stronger overall support structure for the integration of telemonitoring into healthcare delivery. However, regulatory and reimbursement hurdles still existed, and interoperability and data protection remained key operational concerns.

Among the less developed settings, Bulgaria was the country with the least developed regulation framework. The research study identified that Bulgaria still lacks a unified and legally binding definition of telemedicine or RPM. Remote care services are often misclassified as Information and Communication Technologies (ICT) or commercial services rather than clinical interventions, which creates legal ambiguity, uncertainty around liability, and weak institutional confidence. The absence of certification pathways, provider registration mechanisms, reimbursement codes, and clear accountability structures leaves RPM in legal ambiguity. Even though the NHIS offers a potential future anchor for digital care, there is still no national roadmap or coordinated authority dedicated to RPM implementation.

Greece was described by the stakeholders as having an evolving but fragmented regulatory environment. Foundational legal frameworks exist, and the National eHealth Interoperability Framework represents an important policy development, but stakeholders reported that regulation remains narrow and poorly aligned with the practical needs of digital health innovators. A central issue was that existing policy did not clearly address reimbursement, certification, or broader RPM implementation. As a result, many providers and companies were operating in a regulatory grey area, where requirements and responsibilities are not clearly defined, especially when attempting to scale beyond local pilots.

Lithuania was described as being in a developmental regulatory phase. The country had relevant legislation and policy instruments, but participants identified complexity, inconsistency, and a lack of structured support for telemedicine and RPM. Regulatory bottlenecks included difficult certification processes, lack of compensation mechanisms for many telemedicine applications, and insufficient clarity regarding national rules for RPM data handling beyond general data protection requirements.

Spain showed a different pattern: based on the stakeholders’ perspectives, telemedicine was legally permitted and supported by a range of national and EU instruments, but no single dedicated legal framework for remote care existed. As a result, implementation depended heavily on regional organization and local governance arrangements. Portugal was described as having a strong strategic direction through the National Strategic Telehealth Plan, but the Portuguese stakeholder still pointed to gaps in reimbursement and in the practical integration of RPM into national health service workflows.

### 3.3. Interoperability and Health Data Integration

Interoperability and health data integration emerged as an issue in all country contexts. The research study repeatedly identifies fragmented data systems, absent or inconsistently applied standards, and weak integration between RPM tools and existing EHRs as core barriers to adoption. Although HL7 FHIR is highlighted in the stakeholders’ accounts as a key technical standard needed to support scaling, the findings show that standardization remains incomplete and unevenly operationalized.

In order to better understand the nature of interoperability challenges identified across countries, it is important to distinguish between different layers of interoperability [33]. These include syntactic interoperability, referring to the ability of systems to exchange data using standardized formats (e.g., HL7 FHIR); semantic interoperability, which ensures that exchanged data are interpreted consistently and preserve their clinical meaning across systems (e.g., through standardized terminologies and coding systems); and organizational interoperability, which relates to governance structures, data-sharing agreements, and alignment of workflows between institutions. Each interoperability layer has distinguished profiles to indicate the maturity level of each layer. For the syntactic interoperability, which focuses on the structural packaging, coding schemas, and formal formatting of data packets, the profile number indicates, 0.5: Unstructured/Scanned (e.g., paper/flat PDFs), 1.0: Proprietary (e.g., closed vendor file formats), 1.5: Drafted/Pilot-testing (e.g., custom regional schemas), 2.0: Documented/Uniform (e.g., standard XML or JSON), 3.0: Globally Standardized (e.g., universal FHIR, HL7, or ISO profiles). For the semantic profile, which focuses on mapping exact definitions, dictionaries, and codifications so systems understand exactly what data elements mean, the profile number indicates, 0.5: Unmapped/Disconnected (e.g., free text terms), 1.0: Manually Translated (e.g., looking up cross-border equivalents by hand), 1.5: Localized Glossaries (e.g., localized code-sharing registries), 2.0: Codified Databases (e.g., structured using national value sets), 3.0: Universally Decodable (e.g., seamless, automated interpretation using SNOMED-CT or LOINC). Organizational interoperability focuses on alignment between legal mandates, cross-border agreements, operational workflows, and the “human layer” of system integration. Interoperability is categorized with specific profile numbers, ordered in scale in the category set of 0.5/1/1.5/2/3 with number 2.5 unused [34,35]. The definition of each profile number is presented in Table 4. Table 5 outlines the interoperability profiles across the countries that participated in the study. The placement of each country within these descriptive interoperability profiles was based on the combined interview findings and contextual desk research and was finalized through the team-based analytical process described in Section 2.5. The numerical values serve as identifiers for ordered qualitative categories.

The findings of this study suggest that, while progress has been made in adopting syntactic standards, significant gaps remain at the semantic and organizational levels, indicating weaker integration. In many cases, even when technical standards are implemented, differences in data models, lack of harmonized clinical terminologies, and absence of coordinated governance frameworks limit the effective integration of RPM-generated data into EHR and clinical workflows. As a result, interoperability challenges should be understood not only as technical barriers but as systemic constraints requiring coordinated action across technical, organizational, and policy domains [36].

The Belgian case shows that, despite having a relatively advanced ecosystem, one of the most frequently cited barriers was the lack of universal data standards and the difficulty of integrating RPM-generated data into hospital systems. Middleware solutions were described as emerging attempts to bridge this gap, but fragmentation across healthcare institutions and regions remained a persistent obstacle. In Wallonia and Flanders, interoperability was identified not only as a technical issue but also as an organizational and governance challenge.

The Netherlands, according to the stakeholders’ perspective, although more mature among the countries studied, also faced fragmented IT infrastructures and a lack of universal data standards. Interoperability with EHRs was treated as a priority, but not as a solved problem. The Dutch case shows that even in relatively advanced telemonitoring systems, digital maturity does not eliminate integration challenges.

In Bulgaria, the NHIS was described as an important foundational platform, but without systematic support for RPM integration. The research study identified that the NHIS does not currently support real-time data import from RPM devices and does not provide structured interoperability with third-party systems. External RPM systems continue to function in parallel rather than as integrated components of national care coordination. Lack of integration practically results in the lack of remotely generated data visibility in healthcare workflows, while patient-generated data are poorly suited to everyday RPM use.

Greece similarly demonstrated both technical ambition and systemic fragmentation. Interview findings described platforms that integrate multiple diagnostic devices and support data exchange using standards such as HL7 and DICOM, yet stakeholders still emphasized that the national environment lacks a consistently enforced interoperability framework. This issue results in technically capable platforms that exist without being able to connect seamlessly to the broader public health system. The National eHealth Interoperability Framework was acknowledged as a significant step, but its practical implementation remains incomplete.

Lithuania’s findings explicitly addressed data integration. Stakeholders reported that new RPM solutions often struggle to connect with existing EHR systems because of proprietary formats, limited APIs, and slow uptake of standards such as HL7 FHIR. In practice, some integrations still rely on basic formats such as PDF or CSV. Lithuania therefore showed a situation in which promising digital health development coexists with immature data integration pathways. Spain also reported persistent fragmentation, intensified by the decentralized structure of the healthcare system, where autonomous regions maintain distinct digital infrastructures and procurement processes. Even where standards such as HL7 are used, practical interoperability was described as insufficient. The Portuguese stakeholder likewise reported about Portugal, interoperability problems between hospital systems and external platforms, with these data integration issues standing out as one of the main barriers to coordinated RPM implementation.

### 3.4. Implementation Barriers and Ecosystem Readiness

The fourth theme brought together the practical barriers that stakeholders described as influencing whether RPM progresses from innovation to routine use. Across the seven countries, the main barriers were remarkably consistent: unclear reimbursement model/scheme, weak scaling mechanisms, low digital literacy, limited professional readiness, fragmented governance, and insufficient long-term funding. The stakeholder accounts and supporting desk research suggested that settings described as comparatively more developed were associated with stronger infrastructure, clearer frameworks, and greater access to venture capital, whereas less developed settings show innovation potential but remain constrained by reimbursement uncertainty, siloed data systems, and low digital health literacy.

Reimbursement was one of the most recurrent constraints. Belgium’s RIZIV pathway was viewed as difficult even within a relatively developed ecosystem. Bulgaria had no reimbursement pathway for RPM at all, and RPM services were absent from the National Framework Contract. Greece also lacked a consistent or enforceable reimbursement framework, leaving remote monitoring and teleconsultation without reliable public coverage. Lithuania likewise lacked clear reimbursement pathways, while Spain and Portugal, although more institutionally active in digital health, continued to face scaling problems linked to financing and long-term sustainability. These findings indicate that implementation barriers are not limited to early-stage innovators. Even countries with functioning pilots and visible digital infrastructure struggle when payment mechanisms are not aligned with remote care models.

A second major barrier concerned workforce readiness and user acceptance. In Belgium, patient trust, digital literacy, and healthcare professional confidence in data quality were identified as practical barriers to clinical adoption. In Greece, resistance among healthcare professionals was described as being driven by workload pressures, digital illiteracy, and scepticism, especially in the initial stages of adoption. Bulgaria reported similar professional and patient mistrust, compounded by low digital literacy in rural and elderly populations. Lithuania also identified important skills shortages, including not only digital literacy gaps among patients and professionals but also shortages in regulatory, clinical trial, and health economics expertise. Participants from Portugal and Spain reported workforce engagement and training as unresolved issues in both countries.

A third barrier was the weakness of scale-up mechanisms. Across countries, pilots were common, but transition from pilot to sustained implementation was difficult. According to the stakeholders’ perspectives, Bulgaria’s ecosystem was explicitly described as pilot-driven, with no structured route to institutionalization. Greece showed technically mature but geographically limited deployments. Spain highlighted the gap that exists between pilot support and implementation financing. Portugal, despite a substantial number of telemonitoring initiatives, was reported to still face difficulties in integrating these into a coherent and scalable model. Lithuania similarly struggled to connect R&D, clinical validation, procurement, and reimbursement into a continuous innovation pathway.

Ecosystem readiness depended on the degree of collaboration among public authorities, healthcare providers, innovators, research institutions, and investors. Belgium and the Netherlands appeared strongest in this respect because they combined startup ecosystems, research capacity, accelerators, and public–private partnerships. In Greece and Lithuania, ecosystem-building structures existed but were not yet fully translated into national implementation capacity. Bulgaria’s findings were especially clear that the absence of a national coordinator, roadmap, or dedicated RPM governance structure limited institutional confidence and hindered the system-wide use of available innovation potential. Spain and Portugal demonstrated active ecosystems with strong projects and partnerships but also showed that innovation activity alone does not guarantee coordinated health-system uptake.

## 4. Discussion

Across the country contexts examined, stakeholder accounts suggested that RPM implementation was influenced not only by technology availability but also by health-system capacity to integrate, govern, finance, and sustain these solutions. RPM technologies were reported as available and, in several settings, included technically advanced solutions. This aligns with recent evidence suggesting that the impact of RPM depends less on device performance and more on how technologies are embedded within clinical workflows, care pathways, and institutional structures [1,7].

### 4.1. From Digital Innovation to System Transformation

The present country analysis, which was based on a qualitative sample of expert stakeholders rather than nationally representative samples, consistently suggested that despite the availability of RPM technologies, their deployment remains uneven and often limited to pilot settings. This is consistent with broader findings in recent systematic reviews showing that while RPM interventions can improve clinical outcomes and care processes, their effectiveness is highly context-dependent and influenced by implementation conditions [1,8,9].

Companies across Europe are increasingly embedding AI capabilities into RPM solutions, particularly for predictive analytics, risk stratification, AI-assisted diagnostics and clinical decision support. However, the translation of these innovations into routine care remains limited, as companies face challenges related to data access, interoperability with existing health systems, financial affordability and regulatory compliance [37]. As a result, many AI-enabled RPM products remain at pilot or early deployment stages, reflecting the gap between technological development and large-scale implementation, and thus limiting the significant value to health systems and population health. Health care systems could optimize RPM’s impact by focusing on scalability [38].

Within the country contexts examined, the stakeholder accounts and supporting desk research suggested differences in the extent to which existing innovation capacity had been translated into routine, system-level implementation. Even where advanced platforms exist, their integration into routine care is likely to be influenced by alignment with organizational processes, professional practices, and health system incentives. Similar conclusions have been reported in recent qualitative studies, which emphasize that telemedicine adoption is shaped by usability, trust, and workflow integration rather than technological readiness alone [11]. This suggests that RPM should be conceptualized not as a standalone technological intervention, but as a health system innovation, requiring coordinated changes across multiple domains.

### 4.2. Governance, Reimbursement, and the Political Economy of RPM

A recurrent observation across the stakeholder accounts concerned the importance of governance and reimbursement for RPM implementation and scale-up. Participants frequently identified absent, fragmented, or difficult-to-navigate reimbursement arrangements as important barriers. These observations are consistent with policy analyses showing that digital health reimbursement models in Europe remain heterogeneous and frequently provide insufficient support for wider implementation [13].

Even in countries with more advanced digital health ecosystems, reimbursement pathways for RPM remain selective and difficult to navigate [39,40]. In less developed contexts, the absence of reimbursement effectively confines RPM to pilot projects or privately funded initiatives. Taken together, the stakeholder accounts and supporting literature suggest that financial and regulatory arrangements are important conditions affecting digital health adoption.

The broader regulatory environment in Europe is also evolving rapidly. Regulations such as MDR, the AI Act, and the emerging EHDS collectively establish a comprehensive framework governing digital health technologies, data use, and innovation [17,19,27]. However, the findings of this study suggest that the existence of European-level regulation does not guarantee effective national implementation. Instead, legal uncertainty remains a challenge, and the translation of these frameworks into operational policies for certification pathways, procurement rules, and reimbursement mechanisms, amongst others, remains uneven across countries. This gap between regulatory ambition and implementation capacity highlights the need to better understand RPM within the political economy of health systems, where incentives, institutional structures, and policy alignment play a decisive role.

The increasing integration of AI within RPM further accentuates the systemic challenges identified in this study. Across the seven European health systems, RPM platforms are already evolving to incorporate advanced analytics and AI-supported decision-making functionalities. However, their effective and compliant deployment remains highly dependent on the quality, availability, and interoperability of underlying health data. Under the emerging European regulatory framework, particularly the AI Act, many AI-enabled health applications are classified as high-risk systems, requiring robust data governance, traceability, and performance monitoring [19]. In this context, the persistent fragmentation of health information systems and the incomplete implementation of interoperability standards directly affect not only technical performance but also regulatory compliance. As recent research suggests, AI in RPM services has certain challenges like explainability, privacy, and uncertainty [41]. Where RPM data remain poorly integrated into EHRs or are exchanged through non-standardized formats, the reliability and auditability of AI-driven outputs may be compromised. These findings suggest that interoperability and data readiness are foundational conditions for the safe, trustworthy, and scalable deployment of AI-supported remote care within the European regulatory environment.

### 4.3. Interoperability as a Structural Constraint

Interoperability emerged as a persistent and cross-cutting challenge, affecting all countries regardless of their level of digital maturity. The findings show that fragmentation of health information systems, inconsistent use of standards, and limited integration between RPM tools and EHR continue to hinder implementation.

Recent literature supports this observation about interoperability-related challenges and data privacy and security concerns [42]. A 2025 scoping review on telemonitoring interoperability found that, despite increasing adoption of standards such as HL7 FHIR, integration across systems remains incomplete and often dependent on local solutions [14]. This indicates that interoperability is not simply a technical issue but a structural one, involving governance, procurement, and coordination across stakeholders.

It is essential to highlight that the lack of interoperability has implications beyond technical efficiency. It affects clinical workflows, data quality, trust in digital systems, and ultimately the willingness of healthcare professionals to adopt RPM solutions. As highlighted in OECD analyses of health data systems, fragmented data infrastructures limit the ability of health systems to fully leverage digital technologies and constrain the development of data-driven care models [1].

In this context, the EHDS represents a significant opportunity to improve cross-border and national interoperability. However, the findings of this study suggest that its success is likely to be influenced by addressing existing fragmentation at the national level, rather than assuming that European-level frameworks alone will resolve integration challenges.

### 4.4. Limitations of Pilot Implementations

A recurring pattern across all examined countries is the persistence of failure to transition from pilot to routine implementation. Even though RPM initiatives are successfully developed and tested, they often fail to transition into routine, large-scale implementation. This phenomenon has been widely reported in digital health research, where many promising innovations remain confined to pilot phases due to lack of sustainable funding, regulatory clarity, and organizational integration [12,43].

The results show that this pattern is not limited to less developed ecosystems. Even in countries with strong innovation capacity, scaling remains difficult when financial, regulatory, and organizational conditions are not aligned. This suggests that the challenge of scaling RPM is systemic rather than technological.

The concept of ecosystem readiness is therefore critical. Successful digital health implementation requires alignment between infrastructure, governance, financing, workforce capabilities, and stakeholder collaboration [37,39].

### 4.5. Implications for European Digital Health Policy

The findings of this exploratory study are relevant to ongoing European initiatives aimed at strengthening digital health, particularly the EHDS. While the EHDS is expected to improve data access, interoperability, and innovation capacity across Europe, its effectiveness will depend on the ability of national health systems to adopt and operationalize its provisions. Recent commentary in Nature Medicine emphasizes that the EHDS has the potential to significantly enhance cross-border research and innovation, but also warns that disparities in national readiness may limit its impact if not addressed [18]. The results of this study support this concern, showing that countries differ substantially in their governance structures, interoperability capacity, and implementation mechanisms.

This suggests that European policy efforts should not only focus on establishing common frameworks but also on supporting implementation capacity at national and regional levels. To support ecosystems towards implementation capacity, key priorities include developing sustainable reimbursement models for RPM, strengthening interoperability infrastructures, supporting workforce training and digital literacy, and improving coordination between policy, innovation, and healthcare delivery.

The findings support the need for coordinated policy and implementation measures. Sustainable reimbursement models could support RPM to become a viable component of routine care, while regulatory requirements could be translated into clear operational pathways for certification, procurement, liability, and service integration. Greater interoperability with EHR systems may be necessary so that remotely generated information can be incorporated into clinical workflows. Investment in workforce capacity and digital literacy is equally important for strengthening professional confidence, patient trust, and effective use. Finally, closer coordination among healthcare providers, public authorities, innovators, research organizations, funders, and payers could support the creation of practical pathways from successful pilots to wider implementation.

European initiatives such as the EHDS provide an important enabling framework, but their impact will depend on the capacity of national and regional health systems to operationalize their provisions. Particular attention is needed to differences in national readiness so that countries with less established digital health ecosystems can participate effectively in and benefit from European developments. Scaling RPM therefore would benefit from aligned action across policy, financing, infrastructure, workforce development, and healthcare organization rather than the isolated deployment of additional technologies.

### 4.6. Contribution and Limitations

This study contributes to the literature by providing a cross-country exploratory analysis of RPM ecosystems in Europe, grounded in stakeholder perspectives. It complements existing research by shifting the focus from technological evaluation to implementation conditions and system readiness. However, due to the study design, its observations should be interpreted as qualitative assessments of ecosystem maturity development rather than formal country rankings.

At the same time, several limitations should be considered when interpreting the findings of this study. First, the study had a small sample size, and the number of stakeholders differed across countries, with some national contexts represented by a single expert interview. This reflects the combination of purposive expert selection and network-mediated recruitment used in the study, whereby national partners identified stakeholders with substantial expertise and strategic oversight of RPM developments. Recruitment through existing professional networks may nevertheless have favoured stakeholders already connected to the IRHIS ecosystem and may have excluded relevant perspectives beyond those networks. Consequently, the findings should be interpreted as establishing a general overview of the RPM ecosystem development within the respective countries, rather than an exhaustive analysis of initiatives and conditions within a given country.

Second, the study was designed to generate qualitative, policy-oriented insights rather than statistically representative country comparisons. The maturity profiles and ecosystem descriptions presented in this paper therefore represent interpretative syntheses derived from stakeholder perspectives and contextualized with country-specific desk research, rather than formal benchmarking exercises. The availability, scope, and recency of the documentary information also varied across countries. The documentary material was therefore used for contextual interpretation rather than as a standardized evidence base for independent cross-country comparison, and the depth of contextual information may differ between country accounts. As the desk research was intended to provide contextual information, a standardized cross-country data-extraction procedure was not applied. This may have contributed to some variation in the type and level of detail captured across countries. Country-level differences should therefore be interpreted with appropriate caution and within their respective national contexts.

Findings are based on stakeholder perceptions, which may reflect institutional or contextual biases. The interviewers and analysts were members of the IRHIS multidisciplinary team with professional backgrounds in digital health, health informatics, clinical practice, data analysis, healthcare innovation, and digital health policy. Because participants were identified through consortium and professional networks, some members of the research team had professional relationships with participants or prior knowledge of the national ecosystems examined. This insider position may have influenced participant selection, interview interactions, and interpretation of the findings. To reduce the influence of individual assumptions, a common interview guide was used, two researchers conducted the initial coding, a third researcher reviewed the preliminary codes and thematic organization, and the interpretations were discussed with the wider research team. Documentary information was also used to contextualize the stakeholder accounts. Nevertheless, the possibility of network-selection and confirmation bias cannot be excluded.

Despite these limitations, the study offers consistent insights into the systemic factors shaping RPM adoption and highlights key areas for policy and research attention.

### 4.7. Future Research Directions

Future research should extend this exploratory assessment through larger and more balanced samples, particularly in country contexts represented by a limited number of stakeholders. Studies should also include patients, informal caregivers, frontline clinicians, payers, and regulatory authorities to examine how RPM implementation is experienced across different levels of the health system. Longitudinal research will be needed to monitor how national implementation of the EHDS, reimbursement policies, interoperability requirements, and clinical integration pathways affect RPM scale-up over time. Finally, the ecosystem-readiness dimensions identified in this study should be examined prospectively using transparent indicators and repeated data collection before they are used for formal benchmarking or country comparison.

## 5. Conclusions

This exploratory study found that RPM technologies were reported as available across the participating country contexts, while their integration into routine healthcare remained uneven and frequently limited to pilot or locally bounded initiatives. Within the stakeholder accounts and supporting documentary material examined, governance and reimbursement arrangements, regulatory implementation, interoperability, integration with clinical workflows, workforce readiness, and ecosystem coordination were repeatedly identified as conditions influencing the progression of RPM initiatives from experimentation to wider implementation. These findings represent exploratory qualitative observations and should not be interpreted as establishing causal determinants or nationally representative levels of RPM maturity.

## Figures and Tables

**Table 1:** Participant Characteristics (N = 22).

Characteristics	N = 22
Gender	
Male	12
Female	10
Other	0
Place of Operation	
Belgium	3
Bulgaria	4
Greece	3
Lithuania	5
Portugal	1
Spain	3
Netherlands	1
EU	2
Years of experience	
1–10 years	4
10–20 years	11
20+ years	7
Type of Organisation	
Healthcare Providers including hospitals, public health departments	3
Industry including R&D entities, SMEs, Medtech/healthcare clusters	11
Academia including academics and researchers	4
Governmental organisations	4
Occupation	
CEO	7
Head of Department/Director	6
Digital Health Facilitator/Advisor	5
Academic	4

**Table 2:** Synthesis of thematic findings identified from the interviewees.

Themes	Content
Digital infrastructure and RPM technologies	Maturity of digital infrastructure; Types of RPM Technologies; Health system integration
Governance and regulatory frameworks	Governance; Regulatory frameworks
Interoperability and health data integration	Interoperability challenges; Data integration
Implementation barriers and ecosystem readiness	Role of reimbursement, workforce readiness and user acceptance, weakness of scale-up mechanisms, ecosystem readiness

**Table 3:** Types of RPM technologies reported in the examined contexts.

Technology Type	Examples	Contexts in Which Reported
Wearables	smartwatches, patches	All examined contexts
Remote diagnostics	ECG, glucose monitoring devices	BE, NL, ES, PT
Telemedicine platforms	video consults	All
AI decision support	predictive analytics	BE, NL
Implantables	cardiac devices	BE, NL, ES

Abbreviations: BE—Belgium, NL—The Netherlands, ES—Spain, PT—Portugal, GR—Greece, LT—Lithuania, BG—Bulgaria.

**Table 4:** Interoperability rubric and anchor definitions.

Interoperability Layer	Focus	Profile	Anchor Definition/Example
Syntactic interoperability	Structural packaging, coding schemas, and formal formatting of data	0.5	Unstructured/Scanned—e.g., paper records or flat PDFs
		1.0	Proprietary—e.g., closed vendor-specific file formats
		1.5	Drafted/Pilot-testing—e.g., custom regional schemas
		2.0	Documented/Uniform—e.g., standard XML or JSON
		3.0	Globally Standardized—e.g., universal FHIR, HL7, or ISO profiles
Semantic interoperability	Mapping of exact definitions, dictionaries, and codifications so that systems interpret data consistently	0.5	Unmapped/Disconnected—e.g., free-text terms
		1.0	Manually Translated—e.g., cross-border equivalents looked up manually
		1.5	Localized Glossaries—e.g., localized code-sharing registries
		2.0	Codified Databases—e.g., structured national value sets
		3.0	Universally Decodable—e.g., seamless, automated interpretation using SNOMED CT or LOINC
Organizational interoperability	Alignment of legal mandates, cross-border agreements, operational workflows, and the human layer of system integration	0.5	Fragmented/Isolated—e.g., no cross-agency agreements
		1.0	Discretionary/Ad hoc—e.g., informal peer-to-peer cooperation
		1.5	Emerging Governance—e.g., draft memorandums or pilot service-level agreements (SLAs)
		2.0	Legally Aligned—e.g., formalized frameworks and shared policies
		3.0	Optimized Workflows—e.g., fully synchronized cross-border ecosystem

**Table 5:** Interoperability profiles across countries.

Country	Syntactic	Semantic	Organizational	Short Evidence Summary
Belgium	3	2	1.5	Advanced ecosystem, but weak universal standards and difficult hospital integration; governance fragmentation persists.
Netherlands	3	2	2	Priority on EHR interoperability, but fragmented IT infrastructure and unresolved standards limit full integration.
Lithuania	2	1.5	1	Proprietary formats, limited APIs, slow HL7 FHIR uptake; some integrations still depend on PDF/CSV.
Greece	2	1.5	1	Technically capable platforms exist, but national interoperability enforcement and system-wide integration remain incomplete.
Bulgaria	1	0.5	0.5	NHIS is foundational, but real-time RPM import and structured third-party interoperability are missing.
Spain	2	1.5	1	HL7 use exists, but decentralization and regional system fragmentation weaken practical interoperability.
Portugal	2	1.5	1	Hospital-external platform integration remains problematic and hinders coordinated RPM implementation.

## Data Availability

The interview transcripts are not publicly available because the consent arrangements and risk of deductive identification preclude their disclosure. Aggregated, non-identifiable materials may be available from the corresponding author upon reasonable request, subject to applicable data-protection requirements.

## Supplementary Materials

The following supporting information can be downloaded at: https://www.mdpi.com/article/10.3390/healthcare14193147/s1, File S1: Stakeholders Interview Guidelines; File S2: COREQ_Checklist.

## Abbreviations

The following abbreviations are used in this manuscript:

AI	Artificial Intelligence
EHDS	European Health Data Space
EHR	Electronic Health Record
GDPR	General Data Protection Regulation
IVDR	In vitro Diagnostic Regulation
MDR	Medical Device Regulation
NHIS	National Health Information System
RPM	Remote Patient Monitoring
R&D	Research & Development
