# Supplementary material for: Remote Patient Monitoring Across European Health Systems: Policy Insights from a Cross-Country Stakeholder Study
Source: Healthcare (Basel). 2026 Sep 22;14(19):3147. doi: 10.3390/healthcare14193147 (PMC13665366; doi:10.3390/healthcare14193147)
Supplement: Supplementary file 1 [file healthcare-14-03147-s001.zip › Supplementary_File_S2_COREQ_Checklist.pdf]

Supplementary File S2

Consolidated Criteria for Reporting Qualitative Research (COREQ) Checklist

Manuscript: Remote Patient Monitoring Across European Health Systems: Policy Insights from a Cross-Country Stakeholder Study

Reporting note: COREQ was used retrospectively during revision to assess reporting completeness; it did not guide the original study design. Items marked 'not reported' reflect information unavailable in the study record used for this checklist. Items marked 'not undertaken' identify procedures that were not part of the study. Section references are provided now; replace XX page/line entries after final manuscript pagination.

Checklist source: Tong, A.; Sainsbury, P.; Craig, J. Consolidated criteria for reporting qualitative research (COREQ): a 32-item checklist for interviews and focus groups. International Journal for Quality in Health Care 2007, 19(6), 349-357. https://doi.org/10.1093/intqhc/mzm042

No.	COREQ item and guide question	Reporting status and study-specific detail	Location in revised manuscript
**Domain 1: Research team and reflexivity**			
**1**	Interviewer/facilitator<br>Which researcher(s) conducted the interviews?	Interviews were conducted by members of the IRHIS multidisciplinary research team. The manuscript describes the team's relevant expertise; individual interviewer names are not reported.	Section 2.3; Section 4.6
**2**	Credentials<br>What credentials did the interviewer(s) hold?	Specific academic or professional credentials are not itemized. Relevant expertise in RPM, clinical practice, health informatics, data analysis, healthcare innovation, and digital health policy is reported.	Section 2.3; Section 4.6
**3**	Occupation<br>What was the interviewers' occupation at the time of the study?	The interviewers were members of the IRHIS multidisciplinary research team working in digital health, health informatics, clinical practice, data analysis, healthcare innovation, or policy-related fields.	Section 2.3; Section 4.6
**4**	Gender<br>Was the interviewers' gender reported?	Not reported; interviewer gender was not used as an analytical characteristic.	Not reported
**5**	Experience and training<br>What experience or training did the interviewers have?	Relevant multidisciplinary expertise is described. Interviewers completed a structured onboarding process covering the interview guide, interviewing consistency, flexibility, and probing techniques.	Section 2.3
**6**	Relationship established<br>Was a relationship established before study commencement?	Recruitment was facilitated through consortium and professional networks. Some team members had pre-existing professional relationships with participants; this is acknowledged as a potential source of influence.	Section 2.2; Section 4.6
**7**	Participant knowledge of interviewer<br>What did participants know about the researchers and the study?	Participants were informed about the study objectives and provided informed consent. Further details about what participants knew about individual interviewers are not reported.	Sections 2.3 and 2.6
**8**	Interviewer characteristics<br>Which researcher characteristics or assumptions could influence the study?	The reflexivity statement reports the team's professional backgrounds, IRHIS affiliation, network-based recruitment, potential prior relationships, and possible network-selection or confirmation bias.	Section 4.6
**Domain 2: Study design**			
**9**	Methodological orientation and theory<br>Which methodological orientation underpinned the study?	Qualitative, cross-country exploratory design. Interview data were examined using a hybrid deductive-inductive thematic analysis: the initial organization was guided by study objectives and interview-guide domains, while recurring observations within those domains were also recorded.	Sections 2.1 and 2.5

No.	COREQ item and guide question	Reporting status and study-specific detail	Location in revised manuscript
10	Sampling<br>How were participants selected?	Purposive expert selection combined with network-mediated, pragmatic recruitment. Participants were selected for relevant expertise and professional involvement; availability also influenced recruitment. No statistical representation or country-level saturation was claimed.	Section 2.2
11	Method of approach<br>How were participants approached?	Participants were identified and invited through the professional networks of the IRHIS consortium and national partner organizations. The precise communication channel used for each invitation is not reported.	Section 2.2
12	Sample size<br>How many participants were included?	Twenty-two stakeholders participated.	Section 2.2; Table 1
13	Non-participation<br>How many people declined or did not participate, and why?	Not reported; a complete record of refusals or non-participation was not available for this reporting checklist.	Not reported
14	Setting of data collection<br>Where were the interviews conducted?	Interviews were conducted virtually using secure online communication platforms between February and March 2025.	Section 2.3
15	Presence of non-participants<br>Was anyone else present during data collection?	Not reported.	Not reported
16	Description of sample<br>Which participant characteristics were reported?	Table 1 reports gender, country/place of operation, years of experience, organization type, and occupation. The sample included healthcare providers, industry, academia, governmental bodies, and European-level organizations.	Section 2.2; Table 1
17	Interview guide<br>Were questions, prompts, pilot testing, or a guide reported?	A common semi-structured interview guide covered five domains. It was pilot-tested with project partners and refined for wording, sequencing, duration, and probing. The complete guide is provided as Supplementary File S1.	Section 2.3; Supplementary File S1
18	Repeat interviews<br>Were repeat interviews conducted?	No repeat-interview procedure is reported.	Not reported
19	Audio/visual recording<br>Were interviews audio- or video-recorded?	With participant consent, interviews were recorded and subsequently transcribed for analysis.	Sections 2.3 and 2.6
20	Field notes<br>Were field notes made during or after interviews?	Not reported.	Not reported
21	Duration<br>What was the duration of the interviews?	Approximately 45-60 minutes.	Section 2.3
22	Data saturation<br>Was data saturation discussed?	No formal saturation claim was made. The study sought informed expert perspectives and did not aim to establish saturation within each country context.	Section 2.2; Section 4.6
23	Transcripts returned<br>Were transcripts returned to participants for correction or comment?	Not undertaken.	Not undertaken
**Domain 3: Analysis and findings**			

No.	COREQ item and guide question	Reporting status and study-specific detail	Location in revised manuscript
**24**	Number of data coders<br>How many researchers coded the data?	Two researchers performed the initial review and manual coding of all interview transcripts. A third researcher reviewed the preliminary codes and their organization into broader thematic categories. Interpretations were discussed with the wider team and finalized through discussion.	Section 2.5
**25**	Description of the coding structure<br>Was the coding structure described?	Coding was guided primarily by the study objectives and interview-guide domains; recurring observations within these domains were also recorded. Table 2 presents the principal themes and representative codes/subthemes.	Section 2.5; Table 2
**26**	Derivation of themes<br>Were themes identified in advance or derived from the data?	A hybrid deductive-inductive process was used. Initial organization was informed by predefined study and interview-guide domains, while recurring observations were recorded within those domains. A third researcher and the wider research team reviewed and refined the thematic organization.	Section 2.5
**27**	Software<br>Which software was used to manage or analyse the data?	No dedicated qualitative data-analysis software was used. Review and coding were conducted manually.	Section 2.5
**28**	Participant checking<br>Did participants provide feedback on findings?	Not undertaken.	Not undertaken
**29**	Quotations presented<br>Were participant quotations used to illustrate findings?	No. Participants were explicitly assured that contributions would be reported only in aggregated form and that verbatim quotations would not be published. This was important because several participants held senior or publicly identifiable roles and some country contexts had a single participant, creating deductive-identification risk.	Section 2.6; Section 4.6
**30**	Consistency of data and findings<br>Was the relationship between the analysed material and the findings described?	The Results are organized around four principal themes and country-specific observations. Table 2 reports representative codes/subthemes. Verbatim quotations are omitted in accordance with the agreed reporting conditions; this limitation is explicitly acknowledged.	Section 3; Table 2; Section 4.6
**31**	Clarity of major themes<br>Were the major themes clearly presented?	Four principal themes are stated at the opening of the Results, summarized in Table 2, and reported in Sections 3.1-3.4.	Section 3; Table 2
**32**	Clarity of minor themes and diverse cases<br>Were subthemes and variations described?	Representative codes/subthemes are summarized in Table 2. The Results describe similarities and country-specific differences, while the limitations acknowledge uneven participant representation and variation in contextual depth.	Table 2; Sections 3.1-3.4 and 4.6
