# Supplementary material for: Remote Patient Monitoring Across European Health Systems: Policy Insights from a Cross-Country Stakeholder Study
Source: Healthcare (Basel). 2026 Sep 22;14(19):3147. doi: 10.3390/healthcare14193147 (PMC13665366; doi:10.3390/healthcare14193147)
Supplement: Supplementary file 1 [file healthcare-14-03147-s001.zip › Supplementary_File_S1_Stakeholders Interview Guidelines.pdf]

Co-funded by the European Union

Supplementary File S1

Stakeholder interviews guidelines

Manuscript: Remote Patient Monitoring Across European Health Systems: Policy Insights from a Cross-Country Stakeholder Study

IRHIS WP1 - T1.1.3 Stakeholder interviews

How to perform the T1.1.3 Stakeholder Interviews (Guidelines)

Contents

1. Survey questions main thematic axes ... 2
2. Purpose of interview ... 2
3. Methodology ... 2
4. Survey implementation pipeline ... 4
5. Obligatory statements for stakeholder interviews ... 6
6. Provisional timing ... 8
ANNEX 1: IRHIS Final Questionnaire ... 10
ANNEX 2. Interview Analysis Template - Country ... 13

1. Survey questions main thematic axes

The T1.1.3 Stakeholder interviews will be based on 15 questions split on the following 4 thematic axes to gain a comprehensive understanding of the current landscape, challenges, and opportunities in Remote Patient Monitoring (RPM) solutions.

A.1. Advanced remote care innovations
This thematic axis aims to capture insights into the latest advancements in RPM technologies, their applications, and their impact on stakeholders.
A.2. Stakeholders
Understanding the key players in RPM adoption and their roles is critical to ensuring successful implementation. This section focuses on identifying the target groups and the necessary collaboration among stakeholders.
A.3. Challenges, limitations & good practices
This section aims to identify the common barriers to RPM implementation and explore existing good practices that can facilitate smoother integration.
A.4. Recommendations informing the adoption, implementation, and scaling of RPM solutions
This section aims to gather expert recommendations on the future steps needed for widespread RPM adoption and scalability.

At ANNEX 1, you can find the full 'IRHIS Final Questionnaire'.

2. Purpose of interview

We will interview representatives of national and EU federations and leading Medtech clusters and R&D entities from our network **to map advanced remote care innovations, data exchange formats and stakeholders, but also good practices informing on best routes for scaling.** The most advanced remote care monitoring innovations will be put forward and analyzed to draw learnings on their pathway to market and key hurdles faced in the process.

3. Methodology

The stakeholder interview process will employ a qualitative research approach, designed to capture in-depth insights, perspectives, and experiences from key stakeholders involved in Remote Patient Monitoring (RPM) solutions. This methodology focuses on understanding the complexities and nuances surrounding the adoption, implementation, and scaling of RPM technologies.

We will need to conduct **3 – 5 interviews per country**. After the completion of the interviews, **each country will need to complete one Interviews Analysis Template** compiling the key insights from the interviews (see ANNEX 2) and send it to HDHC.

3.1. Survey techniques

To ensure flexibility and inclusivity, multiple survey techniques can be utilized based on stakeholder preferences, availability, and geographical distribution. To enhance inclusivity and clarity, the questionnaire can be *translated into the local languages* of the partner countries involved in the

project or can be translated *ad hoc* (**be careful not to bias the question logic!**). This approach will enhance stakeholder engagement and ensure accurate responses by reducing language barriers.

a) In-person interview (1 hour)

A structured or semi-structured face-to-face interview conducted at a mutually agreed location, typically lasting one hour. This method allows for richer interaction, deeper engagement, and the opportunity to capture non-verbal cues.

Advises

- Ensure a conducive environment that encourages open discussion and minimizes distractions.
- Prepare a structured agenda to optimize time efficiency while allowing room for spontaneous insights.
- Use audio recording (with consent) to ensure accurate data collection.
- Consider logistical challenges such as travel time, scheduling conflicts, and accessibility.

Best for

- Stakeholders with complex insights requiring detailed exploration.
- High-level decision-makers who prefer in-depth discussions.
- Situations where rapport and trust-building are crucial.

b) Video conference interview

A remote, real-time interview conducted via online platforms (e.g., Zoom, Microsoft Teams etc.), providing a cost-effective alternative to in-person meetings while maintaining face-to-face interaction.

Advises

- Ensure a stable internet connection and test equipment beforehand to avoid technical disruptions.
- Provide stakeholders with clear instructions on joining the session and using the platform features.
- Use video conferencing features such as screen sharing for enhanced data presentation.
- Encourage a comfortable, distraction-free environment for participants.

Best for

- International stakeholders or those with travel constraints.
- Situations requiring visual demonstrations or document sharing.
- Time-efficient engagements with busy professionals.

c) Web-form questionnaire

An online survey distributed via a digital platform (e.g., Google forms, Microsoft forms, Survey Monkey etc.) allowing stakeholders to respond at their convenience. This technique provides flexibility and facilitates data collection from a broad audience.

Advices

- Ensure the questionnaire is well-structured, concise, and user-friendly.
- Use clear, jargon-free language to enhance accessibility and comprehension.
- Offer optional sections to allow stakeholders to elaborate if needed.
- Monitor completion rates and follow up with reminders if necessary.

Best for

- Stakeholders with limited availability for real-time interviews.
- Large-scale data collection efforts.
- Standardized responses for easier comparative analysis.

d) Email questionnaire

A structured set of questions sent via email, allowing stakeholders to respond in writing at their own pace. This method provides flexibility but relies on proactive engagement from respondents.

Advises

- Keep the questionnaire concise and structured to encourage completion.
- Reduce technical problems with the document to a minimum. Trial and test before sending.
- Personalize the email to enhance engagement and response rates.
- Offer a deadline for responses while allowing flexibility for extensions if needed.
- Follow up with polite reminders and offer assistance for clarification.

Best for

- Stakeholders who prefer asynchronous communication.
- Scenarios where formal, considered responses are required.
- Respondents with unpredictable schedules.

3.2. Survey implementation considerations

Regardless of the chosen technique, the following considerations will be applied to ensure the success of the survey:

- *Confidentiality and data protection*. Ensure compliance with GDPR and ethical guidelines to protect stakeholder information.
- *Standardized questioning.* Maintain consistency across different techniques to allow for comparability of responses.
- *Stakeholder engagement.* Foster trust and transparency by clearly communicating the purpose, expected outcomes, and benefits of participation.
- *Data analysis.* Responses will be systematically analysed to identify common themes, patterns, and actionable insights.

4. Survey implementation pipeline

The survey implementation will follow a structured pipeline to ensure the efficient collection of qualitative insights from relevant stakeholders. The process includes stakeholder identification, engagement, data collection, and follow-up actions to maximize response rates and data quality.

Step 1: Who should answer?

Representatives of national and EU federations and leading Medtech clusters and R&D entities from IRHIS network.

Step 2: Initial engagement via email (or by-phone invitation /step 3/)

Send a well-structured email to identified experts (or engage via phone call directly) that includes:

- A short introductory text highlighting the purpose, goals, and relevance of the survey.
- Catchy visuals and language to capture attention and generate interest.
- *Option 1.* Invitation for an interview with shared free slots and option to set a other more convenient time.
- *Option 2.* attach the formatted and ready-to-use questionnaire for convenience.
- *Option 3.* link to the web-based questionnaire.
- A leaflet or other material or link to website that provides a visually appealing overview of the project.
- Emphasize on the benefits of participation.

Key considerations

Personalize the email to enhance engagement, and emphasize confidentiality, impact, and the value of their input. Support should be offered in case of questions or clarifications.

Step 3: Follow-up via phone calls

- Explain the survey objectives and expected outcomes.
- Provide an overview of the available participation options (in-person, video call, web form, or email response).
- Align with their preferred schedule and mode of participation.
- Reiterate the benefits of their participation.

Key considerations

Keep the conversation concise yet informative, and do not forget to address any concerns or scheduling conflicts. Offer flexible options to facilitate participation.

Step 4: Reminder strategy

If no response is received:

- *First reminder*. Send a polite follow-up email 3 days after the initial outreach.
- *Second reminder.* If still no response, send another follow-up within 1 week, emphasizing the importance of their contribution.

Key considerations

Keep reminders brief and encouraging, highlight the deadline for responses, and offer additional support if needed.

Step 5: Data collection and processing

Collect responses via the chosen survey technique (in-person, video, web form, email).

- Import structured responses directly into the central spreadsheet or form for consolidation and analysis.
- For interviews conducted in local languages you should translate unstructured text answers and ensure accuracy and consistency before copying responses to the central repository.

Key considerations

Validate and review responses for completeness and address any discrepancies or missing data. If needed a second short call for filling the missing questions is allowed. Do not exceed it more than 15 minutes.

Step 6: Acknowledgment and next steps

Send a thank-you letter to participants upon completion of the survey, including appreciation for their contribution, and a ***call to action*** encouraging them to stay engaged (e.g., "Stay updated, exciting news coming soon"). A link to project updates, newsletters, or upcoming events can be also very suitable.

Key considerations

It is of crucial importance to ***personalize the thank-you message,*** and to avoind any typos or technical mistakes, e.g. mismatching personal data, names, companies etc. Use a positive, forward-looking tone to maintain stakeholder engagement.

Step 7: Follow-up steps

- During team meeting identify bottlenecks and refine the outreach strategy of needed
- Document lessons learned to optimize future stakeholder engagement efforts.

5. Obligatory statements for stakeholder interviews

To ensure consistency, professionalism, and the accuracy of data collection, the following obligatory statements and guidelines must be adhered to during stakeholder interviews. These statements cover key aspects such as interview structure, language, behavior, and methodological considerations.

5.1. Interview structure and conduct

Clear questioning process

- Interviewers must clearly indicate the completion of one question before proceeding to the next to maintain structure and avoid confusion.
- Avoid paraphrasing the questions; they should be presented exactly as formulated to ensure consistency and comparability across interviews.
- Provide the respondent with sufficient time to reflect and respond before moving to the next question.

Feedback collection

- At the conclusion of the interview, request feedback from the respondent regarding the quality of the interview process and methodology.
- Feedback should cover aspects such as clarity of questions, interview duration, and overall experience.
- Document any suggestions for improvement to refine future interviews.

Providing feedback to respondents

- Acknowledge the respondent's contributions and provide general feedback on their input.
- Maintain a neutral stance while offering constructive comments on their responses where appropriate.
- Reinforce the value of their participation and explain how their input will contribute to the project outcomes.

Language use during interviews

- The interview should be conducted in a strictly formal tone to ensure professionalism and maintain a high level of credibility.
- If necessary, some informal language can be used to foster a comfortable environment, but this should be done cautiously and appropriately.
- Maintain a professional yet approachable demeanour to encourage open and honest responses.

Interviewer behaviour and appearance

- Interviewers should exhibit professional behaviour and appropriate attire in all interview formats (in-person, virtual, or phone).
- Maintain a respectful and neutral attitude throughout the interview process.
- Ensure active listening and avoid interrupting the respondent unless necessary to clarify a point.
- Be mindful of cultural sensitivities and adapt communication styles accordingly.

Timing Considerations

- Adhere strictly to the allocated interview duration to respect the respondent's time and commitments.
- Provide a brief overview of the time required at the start of the interview and stick to the agreed schedule.
- If additional discussion is required, offer to schedule follow-up meetings rather than extending the current session excessively.

5.2. Important warnings

Warning 1. Avoid paraphrasing the questions for better understanding by the respondent. Each question must be asked verbatim to ensure standardization and data integrity across all interviews.

Warning 2. Focus groups are not recommended for conducting interviews. Organizing interviews in group settings poses a significant risk of biasing the results, making them potentially misleading and unreliable. Individual interviews should always be prioritized to ensure data accuracy and authenticity.

6. Provisional timing

Question No.	Provisional Timing (minutes)	Key Considerations
**1**	3-4	The question contains three sub-questions; ask them sequentially. Consider replacing "region" with "country" for national experts.
**2**	1-3	Keep responses concise and focus on specific medical domains.
**3**	2-3	Ensure clear identification of geographical regions benefiting from RPM innovations.
**4**	1-3	Clarify technical terms related to data exchange formats for better understanding.
**5**	2-3	Focus on identifying key target groups concisely.
**6**	2-3	Clarify collaboration roles without overwhelming the respondent.
**7**	3-5	Be prepared for detailed responses; consider examples of challenges faced.
**8**	2-3	Ask about connectivity and interoperability separately if needed.
**9**	2-3	Ensure a focus on obstacles that may hinder RPM initiatives.
**10**	2-3	Clarify "factors" related to personal motivation sources (e.g., family, professional goals).
**11**	3-5	Explore good practices thoroughly but within time limits.
**12**	2-3	Encourage concise examples of existing guidelines supporting RPM.
**13**	3-5	Discuss roadmap development while keeping responses goal-oriented.
**14**	2-3	Identify key drivers for RPM adoption concisely.
**15**	2-3	Ask for additional recommendations in a focused manner.

Total estimated interview duration

50 - 55 minutes

Please ensure no long question exceeds 5 minutes and shorter questions stay within 3 minutes.

Additional notes for interviewers

- Stick to the **proposed timing** to avoid exceeding the overall interview duration. Politely guide the respondent back to the topic if discussions drift.
- Be prepared to provide further clarifications where needed, especially for terms that may cause confusion (e.g., "factors," "region").
- Ensure a natural conversation flow while maintaining a structured approach.
- Allow for brief pauses in web-conference interviews to accommodate potential delays due to connectivity issues.

- In in-person interviews, **observe body language** to adjust the pacing and engagement level accordingly.

ANNEX 1: IRHIS Final Questionnaire

Personal data to be collected

Name:<br>Organization:<br>Position:<br>Email:<br>City:<br>Country:<br>Federation (National/ European):<br>Type of organization (R&D entity, Medtech cluster):<br>Field of expertise:<br>Years of RPM experience:

THEMATIC AXES

Advanced remote care innovations

1. Could you outline the key innovations in Remote Patient Monitoring (RPM) currently being implemented within your region? Which are their detailed features? Please, specify their value and their impact on stakeholders. (*e.g. new technologies introduced, new legislation, new funding tools, new companies)*

2. In which specific medical domains are these RPM innovations being applied? (*e.g. musculoskeletal, neurological, critical care)*

3. Which geographical regions are benefiting from the implementation of these innovations?

4. What data exchange standards or/and architecture models exist in RPM solutions? Which data exchange formats do they use (concerning both software and hardware etc.)? *(e.g. interoperability framework in the market, HL7, Continua https://www.pchalliance.org/about-continua)*

Stakeholders

5. Which are the target groups to which your RPM solutions are addressed to? *(e.g. patients, healthcare professionals, management department in healthcare settings, insurance companies etc.*

6. Who should collaborate on the effective adoption and implementation of RPM solutions? *(e.g. healthcare professionals, developers, technical staff, regional health authorities, public authorities etc.)*

Challenges, limitations & good practices

7. What are the main challenges you typically encounter when adapting to new therapy with technology, and how do these challenges influence your decision-making or approach to integrating these innovations into your work or daily life?

8. What kind of challenges may arise concerning the connectivity and interoperability of the data collected and exchanged by RPM solutions?

9. What are the main obstacles that may prevent the adoption and implementation of RPM initiatives?

10. Does the existing regulatory framework at national and European level assist or hinders the adoption and implementation of RPM initiatives? / What role does the national security system play in the adoption and implementation of RPM initiatives in the EU (ethics regulatory framework, cybersecurity, protection of critical health infrastructures, safeguarding sensitive medical data etc.)?

11. What factors most strongly influence your work, and where do you typically find the motivation or inspiration to embrace change and innovation?

12. What are the Good Practices or Guidelines that support or are implemented in your RPM solutions? How do they work?

Recommendations informing the adoption, implementation and scaling of RPM solutions

13. What steps could be taken to develop a comprehensive roadmap, concerning the adoption of RPM solutions in Europe in terms of state of development, degree of acceptability by key stakeholders?

14. Who should take the lead in driving these initiatives?

15. Do you recommend any additional good practices or guidelines to support the adoption, implementation, and scaling of RPM solutions?

ANNEX 2. Interviews Analysis Template - Country

1. Interviewees Demographics

No of interviewees	
Type of interview (in person, online)/ or self-administered questionnaire	
Gender of Interviewees	
Organizations & Positions	
Federations (National/European)	
Type of Organizations (R&D Entity, MedTech Cluster, etc.)	
Fields of Expertise	
Years of RPM Experience	

2. Thematic Axes for Analysis

A. Advanced Remote Care Innovations

(List a Summary of Key Innovations in Remote Patient Monitoring (RPM) / Detailed Features / Value and Impact on Stakeholders / Medical Domains of Application Geographical Reach / Data Exchange Standards, Architecture Models, Data Formats)

B. Stakeholders

(List Target Groups for RPM Solutions / Key Collaborators for Effective Adoption and Implementation of RPM Solutions such as patients, healthcare professionals, management teams, insurers, healthcare professionals, tech developers, public authorities etc.)

C. Challenges, Limitations & Good Practices

(Describe Challenges in Adapting to New Therapies with Technology / Connectivity and Interoperability Challenges, e.g. technical or regulatory barriers related to data management)

(List main *<u>Obstacles</u>* *to RPM Adoption and Implementation e.g., funding, resistance to change, lack of infrastructure)*

(Describe *<u>the Impact</u>* *of Regulatory Frameworks / National and EU-Level Regulations /Role of National Security Systems)*

(Describe Influential Factors and Sources of Motivation / *<u>Good Practices or Guidelines</u>* *supported or implemented in RPM Solutions / Successful strategies and operational guidelines currently in use)*

D. Recommendations for Adoption, Implementation, and Scaling of RPM Solutions

(Describe any *<u>Proposed Steps</u>* *for Developing a Comprehensive Roadmap for the Adoption of RPM Solutions in Europe/ Information about best positioned stakeholders to lead)*

(Include any Additional Good Practices or Guidelines suggested)

3. Conclusions and Final Recommendations

(Summarize key points, and critical observations from the interviews / Include key recommendations and suggestions derived from stakeholder responses)

